# Characterization of Some *Cichorium* Taxa Grown under Mediterranean Climate Using Morphological Traits and Molecular Markers

**DOI:** 10.3390/plants12020388

**Published:** 2023-01-13

**Authors:** Ahmed M. El-Taher, Hala A. Elzilal, Hany S. Abd El-Raouf, Emad Mady, Khalid S. Alshallash, Rasha M. Alnefaie, Ehab M. B. Mahdy, Osama G. Ragab, Elhassan A. Emam, Ibrahim A. Alaraidh, Timothy O. Randhir, Mohamed F. M. Ibrahim

**Affiliations:** 1 Department of Agricultural Botany, Faculty of Agriculture, Al-Azhar University, Cairo 11884, Egypt; 2 Department of Science and Technology, College of Ranyah, Taif University, P.O. Box 11099, Taif 21944, Saudi Arabia; 3 Biology Department, University College, Taif University, Turaba 29731, Saudi Arabia; 4 Horticulture Department, Faculty of Agriculture, Al-Azhar University, Cairo 11884, Egypt; 5 Department of Environmental Conservation, University of Massachusetts, Amherst, MA 01003, USA; 6 College of Science and Humanities-Huraymila, Imam Mohammed Bin Saud Islamic University (IMSIU), Riyadh 11432, Saudi Arabia; 7 Biology Department, Faculty of Science Albaha University, Albaha 65779, Saudi Arabia; 8 National Gene Bank (NGB), Agricultural Research Center (ARC), Giza 12619, Egypt; 9 Botany and Microbiology Department, Faculty of Science (Boys Branch), Al-Azhar University, Cairo 11884, Egypt; 10 Department of Microbiology and Cell Biology, Indian Institute of Science, Bangalore 560012, India; 11 Department of Botany and Microbiology, Faculty of Science, King Saud University, Riyadh 11362, Saudi Arabia; 12 Department of Agricultural Botany, Faculty of Agriculture, Ain Shams University, Cairo 11566, Egypt

**Keywords:** chicory, morphological description, molecular authentication, SEM

## Abstract

The verification of taxonomic identities is of the highest significance in the field of biological study and categorization. Morpho-molecular characterization can clarify uncertainties in distinguishing between taxonomic groups. In this study, we characterized five local taxa of the genus *Cichorium* using morphological and molecular markers for taxonomic authentication and probably future genetic improvement. The five *Cichorium* taxa grown under the Mediterranean climate using morphological traits and molecular markers showed variations. The examined taxa showed a widespread range of variations in leaf characteristics, i.e., shape, type, texture, margin, and apex and cypsela characteristics i.e., shape, color, and surface pattern. The phylogenetic tree categorized the *Cichorium intybus* var. *intybus* and *C. intybus* var. *foliosum* in a single group, whereas *C. endivia* var. *endivia* was grouped separately. However, *C. endivia* var. *crispum* and *C. endivia* subsp. *pumilum* were classified as a cluster. The recorded variance between classes using the molecular markers SCoT, ISSR, and RAPD was documented at 34.43%, 36.62%, and 40.34%, respectively. Authentication using molecular tools proved the usefulness of a dichotomous indented key, as revealed by morphological identification. The integrated methodology using morphological and molecular assessment could support improved verification and authentication of the various taxa of chicory. It seems likely that the Egyptian chicory belongs to *C. endivia* subsp. *pumilum*.

## 1. Introduction

The Asteraceae plants, the world’s largest and most diversified flowering plant family, containing 1600–1700 genera and about 25,000–35,000 species, is subdivided into 13 subfamilies, including Barnadesioideae, Cichorioideae, and Asteroideae [1,2]. The phylogeny and diversifications of the Asteraceae family have been impeded by the absence of extensive research into several genera and representative species. It is known that chicory and endive were grown in ancient Egypt, and currently they represent important components in Mediterranean diets. Both are now cultivated worldwide, especially in the Mediterranean basin. The subfamily Cichorioideae has the tribe Lactuceae, which contains the *Cichorium* genus. This genus includes *Cichorium intybus* L. (chicory) and *C. endivia* L. (endive), representing two distinct species recognized according to provenance. The European flora is referred to the *C. intybus, C*. *spinosum*, and *C. endivia* species, subdividing the latter into two subspecies; one is cultivated (subsp. *Endivia*) (cultivated) and the other is wild (subsp. *divaricatum*) as detailed in Tutin et al. [3]. The reference to the Italian flora combines three wild species of *C. intybus* and *C. spinosum*, with the botanical variety *glabratum* (Presl) Fiori, along with *C. pumilum* and *C. endivia* in a single cultivated species [4]. Asteraceae in Egypt includes one wild species of *Cichorium* [5].

Seven *Cichorium* species were characterized morphologically by a revision of Bedarff [6]. *C. endivia* and *C. intybus* split further into two subspecies; C. *endivia* contained subsp. *endivia* and subsp. *divaricatum*, while *C. intybus* was subdivided into subsp. *intybus* and subsp. *glabratum*. This diverges from the Flora Europaea database, Royal Botanical Gardens, which indicates only three subspecies of *C. intybus*: subsp. *foliosum* (Hegi) Janch., subsp. *glabratum* (C. Presl) Arcang., and subsp. *sativum* (Bisch.) Janch. 

The integration between morphological characteristics and molecular findings characterized the two cultivated and well-known *Cichorium* species, *C. intybus* and *C. endivia*, as well as the wild species of *C. pumilum* and *C. spinosum* [7]. In addition, two other species were not found in Europe, *C. calvum* Schultz-Bip and *C. bottae* Defl. *C. calvum* is prevalent in hot and dry areas of Southwestern Asia and the Middle East., while *C. bottae* is found only in Saudi Arabia and Yemen. Finally, in Italian flora, three species of the genus were documented: *C. endivia*, with the two subspecies *pumilum* (Jacq) Cout. and *endivia* Hegi, *C. intybus*, altogether with the two subspecies *glabratum* (C. Presl) Arcang., and *intybus*, and *C. spinosum* [8]. 

Recently, Bartolucci et al. [9] published an updated list of the vascular flora native to Italy, including the genus *Cichorium* with three species: *C. endivia* L. subsp. *pumilum* (Jacq.) Cout., *C. intybus* L., and *C. spinosum* L.

*Cichorium intybus* could be classified into four varieties based on the purpose and usage for which it was cultivated [10]. *C. intybus* subsp. *intybus* can be categorized into five major groups, including all cultivated forms of chicory [11,12]. Apart from wild accessions, the first group refers to the var. *foliosum*, which contains the Witloof chicory. It is suggested that the Witloof chicory (*Cichorium intybus* L. var. *foliosum*, syn. Belgian endive, chicon) taken from Magdeburg roots should be classified under var. *sativum* with all the other root types; many reviews referred to Witloof as having botanical characteristics of the variety *foliosum* [7,12,13,14]. 

Jana and Mukherjee [15] described the testa structure of *C. endivia* and *C. intybus*. *C. intybus*, a biennial species, which has a characteristic inflorescence (capitulum) unique in the family containing 15–25 hermaphrodite flowers with an involucre protecting the receptacle. Every flower possesses a gamopetalous and ligulate corolla with five filamentous stamens. Stamens form a column with fused anthers surrounding the pistil with a bifid stigma [16].

Molecular investigations have been efficiently employed to evaluate the identification and relationship of various taxa. Numerous PCR-based molecular markers are widely used such as random amplified fragment DNA (RAPD), inter-simple sequence repeats (ISSR), and start codon target (SCoT) applying in various taxa [17,18,19,20], which can be used to estimate genetic relationships and variations between and within taxa [21,22,23]. A limited number of molecular studies have integrated with morphological differentiations in relation to biodiversity, identification, and taxonomy in the taxa of chicory. The genetic identification and differentiation of a taxon are applied in various species such as *Brassica* [24], broccoli and cauliflower [25], lettuce and Jew’s mallow [26], and tomato [27]. In *Cichorium*, various molecular markers were applied [7,28,29,30,31,32,33,34,35,36,37]. Some of these markers have been used for genetic map construction [31,32], genetic variation [33,34], gene flow [35], population structure [37], and hybridization [36].

The Egyptian chicory (“Sreece” according to its local Arabic name) belongs to the *C. endivia* sub-species *pumilum* in the Asteraceae family. However, several published articles from Egypt still incorrectly assume that the Egyptian chicory belongs to the *C. intybus* species [38,39,40]. The integrated approach using micro-macro morphological assessment and molecular techniques could help to improve authentication and verification of the species and taxa. With the lack of extensive sampling of several genera and representative species, the family’s phylogeny and diversifications have been hampered by the limited availability of research covering species and varietal diversity, along with the high resemblance among species. 

This study highlights the morphological and molecular characteristics of five taxa of the genus *Cichorium* through assessment and discrimination to determine the relationships among taxa. The micro- and macro-morphological data generated a relationship among the taxa of *Cichorium,* while the PCR-based markers provided information on *Cichorium*’s genetic relatedness. The goal of this study is to present analyses to accurately classify the studied chicory species. In order to resolve the classification ambiguity, we offer a robust argument by documenting morphological and genetic differences between the *C. endivia* and *C. intybus* species of chicory.

## 2. Results

### 2.1. Morphological Characterization

#### 2.1.1. Leaf Morphology 

**Figure 1:**
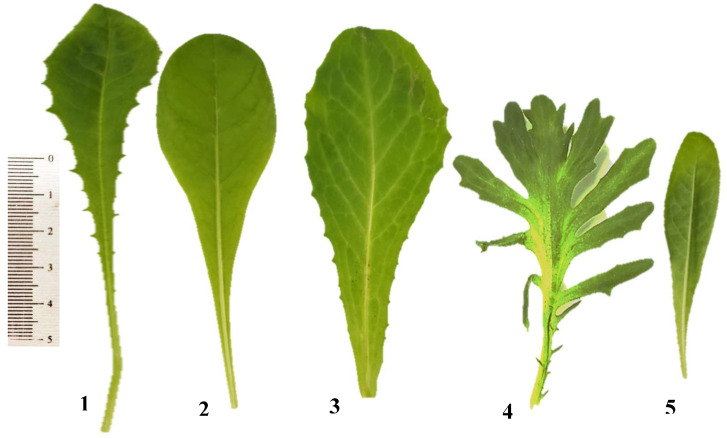
Leaf shapes of Cichorium taxa. (**1)**: C. intybus var. intybus; (**2**): C. intybus var. foliosum; (**3**): C. endivia var. endivia; (**4**): C. endivia var. crispum; (**5**): C. endivia subsp. pumilum.

The leaves of the studied *Cichorium* taxa have the following characteristics in common: they are simple, reticulate, pinnate, glabrous, and exstipulate, and have symmetrical epulvinate bases (Table 1 and Figure 1). The shape of the studied leaves is obovate in *C. endivia* var. *crispum*, oblancelate in *C. endivia* subsp. *pumilum,* and spathulate in *C. intybus* var. *intybus, C. intybus* var. *foliosum,* and *C. endivia* var. *endivia*. The leaves are petiolate in *C. intybus* var. *intybus*, *C. intybus* var. *foliosum,* and *C. endivia* subsp. *pumilum* but, they are sessile in *C. endivia* var. *endivia* and *C. endivia* var. *crispum*. The leaf blade is simple in all of the studied plants, except *C. endivia* var. *crispum,* whose leaf blade is pinnately lobed. Margins of the leaves are dentate in most of the examined taxa, as in *C. intybus* var. *intybus, C. endivia* var. *endivia,* and *C. endivia* var. *crispum,* but they are finely dentate in *C. intybus* var. *foliosum* and *C. endivia* subsp. *pumilum*. All of the investigated plants have obtuse leaf apex shapes, as in *Cichorium*. The midrib is distinct in all plants except *C. endivia* var. *crispum*. Generally, within each taxon, remarkable variability was not noticed.

#### 2.1.2. Cypsela Morphology

**Figure 2:**
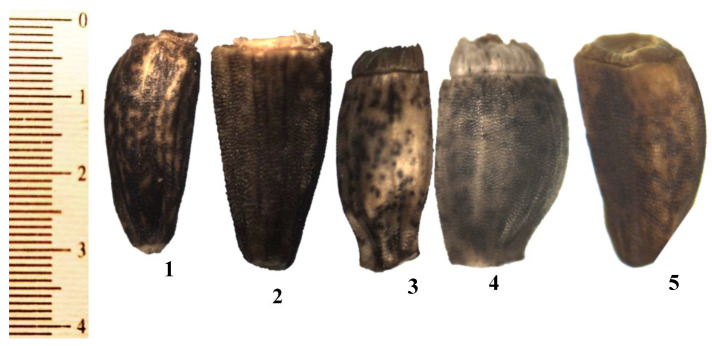
Light micrographs of cypsela and pappus in *Cichorium*: (**1**): *C. intybus* var. *intybus*; (**2**): *C. intybus* var. *foliosum*; (**3**): *C. endivia* var. *endivia*; (**4**): *C. endivia* var. *crispum*; (**5**): *C. endivia* subsp. *pumilum*.

**Figure 3:**
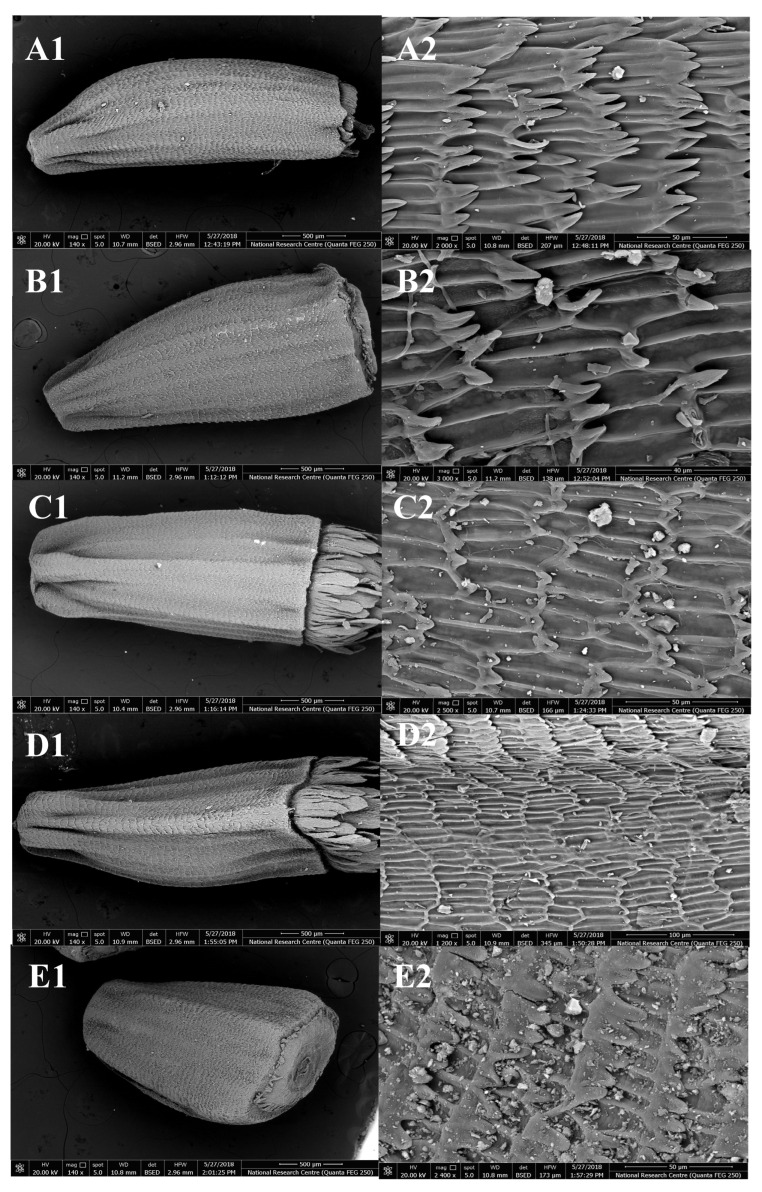
SEM micrographs of cypsela and pappus in *Cichorium*: (**A1**,**A2**): *C. intybus* var. *intybus*; (**B1**,**B2**): *C. intybus* var. *foliosum*; (**C1**,**C2**): *C. endivia* var. *endivia*; (**D1**,**D2**): *C. endivia* var. *crispum*; (**E1**,**E2**): *C. endivia* subsp. *pumilum*.

Details of the micro-morphological characteristics of the cypsela of *Cichorium* are presented in Figure 2 and Figure 3 and Table 1. The cypsela surface is smooth and sometimes rough, and the shape of the hilum is circular. The anticlinal wall shape is straight, and there is pappus persistence in all studied taxa. Fruits of *Cichorium* are cypsela (achene). The shape is oblong in *C. intybus* var. *intybus*, *C. endivia* var. *endivia,* and *C. endivia* var. *crispum,* but oblong-obovate in *C. intybus* var. *foliosum* and *C. endivia* subsp. *pumilum*. The color ranges from creamy and shiny to black: *C. intybus* var. *intybus* is creamy, *C. intybus* var. *foliosum* and *C. endivia* var. *endivia* range from creamy shiny to dark brown and creamy, *C. endivia* var. *crispum* ranges from pale, creamy, and shiny to creamy with a black point, and *C. endivia* subsp. *pumilum* is creamy shiny with a few dark brown spots. The surface pattern of *C. intybus* var. *intybus* and *C. intybus* var. *foliosum* is sulcate papillate, that of *C. endivia* var. *endivia* is weak sulcate papillate, that of *C. endivia* var. *crispum* is ruminate sulcate, and that of *C. endivia* subsp. *pumilum* is weak ruminate-sulcate papillate. The anticinal wall is raised in *C. intybus* var. *intybus* and *C. intybus* var. *foliosum,* and slightly raised in *C. endivia* var. *endivia, C. endivia* var. *crispum,* and *C. endivia* subsp. *pumilum.* Periclinal walls are concave in *C. intybus* var. *intybus* and *C. intybus* var. *foliosum*, but only slightly concave in *C. endivia* var. *endivia, C. endivia* var. *crispum,* and *C. endivia* subsp. *pumilum*. Pappus bristles may be scabrous scales in *C. intybus* var. *intybus* and *C. intybus* var. *foliosum* and paleaceous scales to form a crown in *C. endivia* var. *endivia, C. endivia* var. *crispum,* and *C. endivia* subsp. *pumilum*. Pappus length is short in *C. intybus* var. *intybus*, *C. intybus* var. *foliosum,* and *C. endivia* subsp. *pumilum,* but long in *C. endivia* var. *endivia* and *C. endivia* var. *crispum*.

#### 2.1.3. Epidermal Characteristics

**Figure 4:**
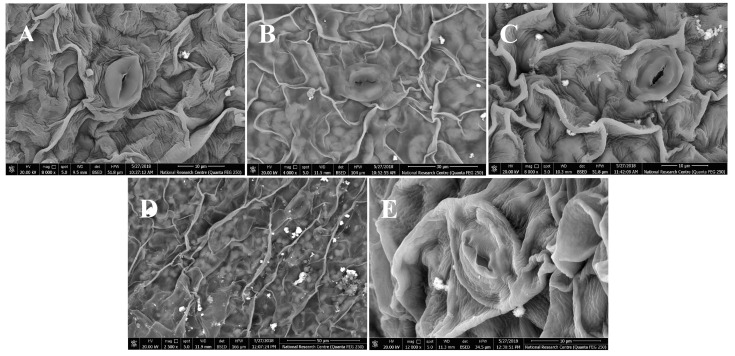
SEM micrographs of stomata level and ornamentation in *Cichorium*: (**A**): *C. intybus* var. *intybus*; (**B**): *C. intybus* var. *foliosum*; (**C**): *C. endivia* var. *endivia*; (**D**): *C. endivia* var. *crispum*; (**E**): *C. endivia* subsp. *pumilum*.

**Figure 5:**
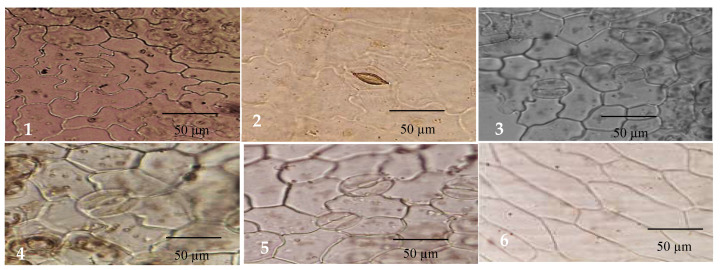
Light micrographs of stomatal types in *Cichorium*: (**1**): Anisocytic stomata; (**2**): Anomocytic stomata; (**3**): Tetracytic stomata; (**4**): Stephanocytic stomata; (**5**): Associated stomata with middle lamella; (**6**): Straight epidermal cell wall.

**Figure 6:**
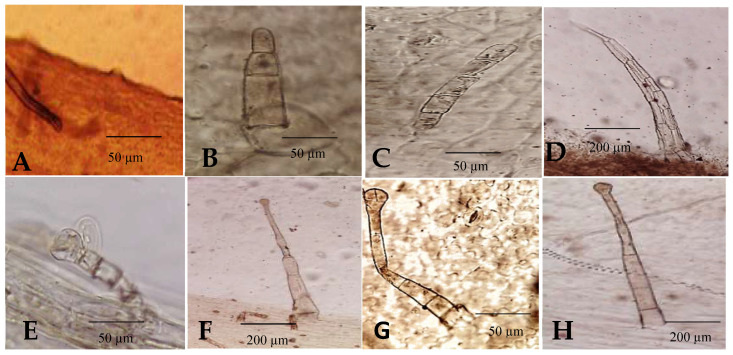
Light micrographs of trichomes in *Cichorium*: subfigures (**A**–**D**): non-glandular, subfigures (**E**–**H**): glandular.

In this study, five taxa of *Cichcorium* were investigated for their epidermal characters and trichomes, in order to determine the essential characters for identification and discrimination between the studied taxa. The different micro-morphological characters are presented in Table 2 and Figure 4, Figure 5 and Figure 6. The epidermal cell wall is straight in two taxa and sinuous in three taxa. Stomata leveling are recorded in three types: superficial, in *C. intybus* var. *foliosum* and *C. endivia* subsp. *pumilum*, semi-depressed, in *C. endivia* var. *endivia* and *C. endivia* var. *crispum*, and depressed, in *C. intybus* var. *intybus* (Figure 4).

The stomata can be categorized into five types, i.e., anisocytic stomata, in *C. intybus* var. *intybus*, *C. endivia* var. *endivia,* and *C. endivia* var. *crispum*, anomocytic stomata, present in all studied taxa, tetracytic stomata, in *C. intybus* var. *intybus, C. endivia* var. *crispum,* and *C. endivia* subsp. *pumilum*, stephanocytic stomata, in *C. intybus* var. *intybus*, *C. intybus* var. *foliosum, C. endivia* var. *crispum,* and *C. endivia* subsp. *Pumilum*, and associated stomata with middle lamella, in *C. endivia* var. *endivia* and *C. endivia* subsp. *pumilum* (Table 2 and Figure 5). Minimal intra-individual variability among stomata was noticed.

#### 2.1.4. Trichomes

The following trichomes have been found on the leaves of the studied species:

- Non-glandular
  1. Papillae (Figure 6A): *C. intybus* var. *intybus* and *C. endivia* var. *endivia*.
  2. Multicellular hairs with heterogeneous cells (Figure 6B): *C. intybus* var. *foliosum*.
  3. Multicellular hairs with homogeneous cells (Figure 6C): *C. endivia* subsp. *pumilum*.
  4. Shaggy hair (Figure 6D): *C. intybus* var. *intybus* and *C. endivia* var. *endivia* and *C. endivia* subsp. *Pumilum*.
- Glandular
  1. Multicellular uniseriate stalk and unicellular head with homogeneous cells (Figure 6E): *C. endivia* var. *crispum*.
  2. Multicellular uniseriate stalk and unicellular head with heterogeneous cells (Figure 6F): *C. intybus* var. *intybus*, *C. endivia* var. *crispum,* and *C. endivia* subsp. *pumilum*.
  3. Multicellular uniseriate stalk and bicellular head with homogeneous cells (Figure 6G): *C. endivia* var. *endivia*.
  4. Multicellular uniseriate stalk and bicellular head with homogeneous cells (Figure 6H): *C. intybus* var. *foliosum*.

#### 2.1.5. Morphological Identification

The data recorded in Table 1 and Table 2 were applied to create the following bracketed key for the five taxa of *Cichorium,* which can help confirm their identity.
1.The leaf shape is spathulate, the anticlinal wall shape is straight, the anticlinal walls are raised, the periclinal walls are concave, and the pappus bristles are scabrous scales.2     The leaf shape is obovate or oblancelate, the anticlinal wall shape is straight, the anti-clinal walls are slightly raised, the periclinal walls are slightly concave, the pappus bristles are paleaceous scales to form a crown.32.The leaf margin is dentate, the shape of the cypsela is oblong, the stomata leveling is depressed, papillae are present, shaggy hair is present, there is a multicellular uniseri-ate stalk, and a unicellular head and heterogeneous cells are present.*C. intybus* var. *intybus*
     The leaf margin is fine dentate, the shape of the cypsela is oblong obovate, the stomata leveling is superficial, papillae are absent, shaggy hair is absent, there is a multicellular uniseriate stalk, and a unicellular head and heterogeneous cells are ab-sent.*C. intybus* var. *foliosum*
3.The leaf shape is obovate, the leaf type is pinnately lobed, the midrib color is green, the radical leaf is absent, the epidermal cell wall is sinuous, there is a multicellular uniseri-ate stalk, and a unicellular head with homogeneous cells are pre-sent.*C. endivia* var. *crispum*
     The leaf shape is spathulate or oblancelate, leaf type is simple, midrib color is white, radical leaf is present, epidermal cell wall is straight, with multicellular uniseriate stalk, and unicellular head with homogeneous cells absent.44.The leaf shape is spathulate, the petiole is absent, the shape of the cypsela is oblong, the surface pattern is weak sulcate papillate, the stomata level is semi-depressed, the epi-dermal sculpture is reticulate, and papillae are present.*C. endivia* var. *endivia*
     The leaf shape is oblancelate, the petiole is present, the shape of the cypsela is oblong obovate, the surface pattern is weak ruminate sulcate papillate, the stomata level is superficial, the epidermal sculpture is striate, and papillae are absent.*C. endivia* subsp. *pumilum*



### 2.2. Genetic Characterization and Polymorphism

**Figure 7:**
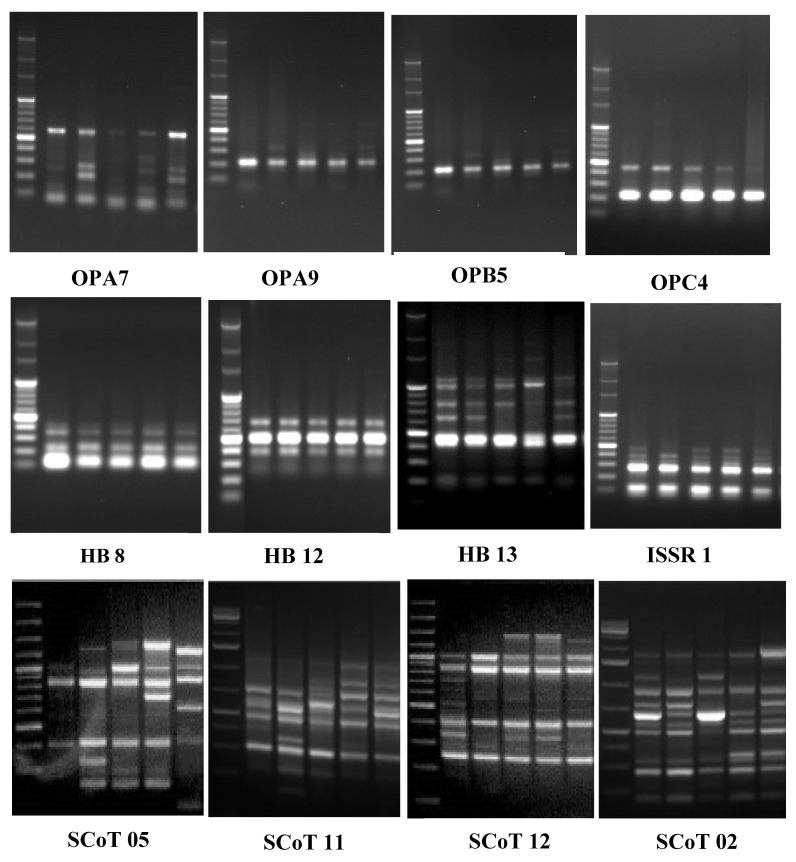
Band profiles of SCoT, ISSR, and RAPD markers. Lane 1: DNA ladder (100 bp), Lanes 2 to 6: Taxa 1 to 5 (From left to right).

The three studied PCR-based markers, RAPD, ISSR, and SCoT, demonstrated high and significant polymorphism and determined the taxon relationship (Figure 7). Table 3 presents total, monomorphic, polymorphic, and unique bands, along with the percent of polymorphism calculated. The thirty primers revealed a total of 186 bands with an average of 6 bands per primer. The polymorphism summed up an average of 58%. A wide range has revealed polymorphism among three markers, as the following of 46% for ISSR, 62% for RAPD, 66% for SCoT-PCR. Primers OPA18 and SCoT-04 generated the highest polymorphism of 100%, while HB-12, SCoT-13, and SCoT-14 produced 25%, 16.7%, and 0%, respectively. Unique bands summed up 17 amplicons of which 5 bands are by RAPD, 7 bands by ISSR, and 5 bands by SCoT marker. The marker UBC807 recorded the highest number of unique bands (three bands). This variability can be used to differentiate the studied taxa.

### 2.3. Morphological and Genetical Relationship and Correlation among Cichorium Taxa

**Figure 8:**
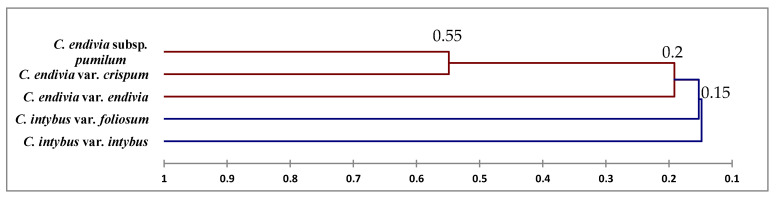
Dendrogram of the relationships between the five taxa of *Cichorium* based on 33 morphological characters using UPGMA analysis.

The morphological characters were analyzed numerically using the clustering method for identification and differentiation between them, a dendrogram using the UPGMA cluster analysis, is presented in Figure 8. The cluster analysis showed that species were grouped into two major clusters. Cluster I consisted of two taxa *Cichorium intybus* var. *intybus* and *Cichorium intybus* var. *foliosum.* Cluster II comprised three taxa *Cichorium endivia* subsp. *pumilum*, *Cichorium endivia* var. *crispum,* and *Cichorium endivia* var. *endivia*.

**Figure 9:**
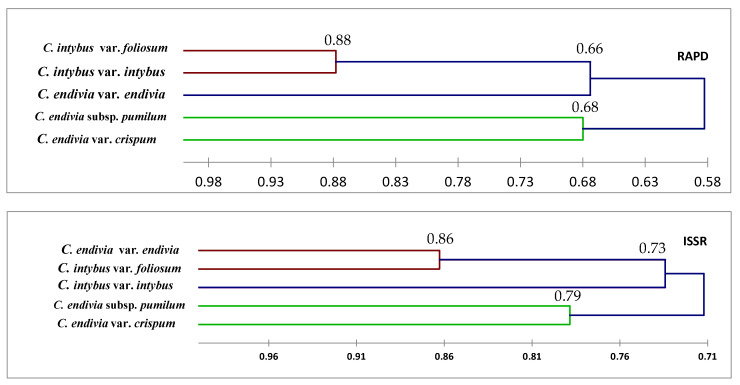

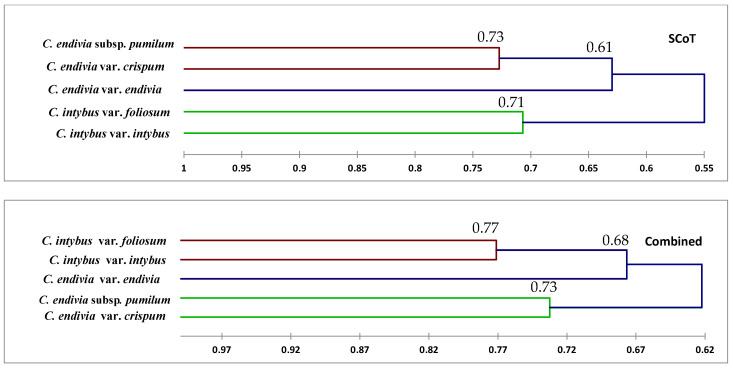
Dendrograms classified the five taxa of Cichorium by three molecular markers using UPGMA analysis.

Genetic similarity was built as a dendrogram following Nei and Li coefficient using the UPGMA cluster analysis (Figure 9). The taxa are divided into two main groups, except the ISSR markers, which reveal a different result among taxa. The *C. endivia* var. *crispum* and *C. endivia* subsp. *pumilum* fell into the group, while *C. intybus* var. intybus and *C. intybus* var. *foliosum* fell into subgroup. The ISSR investigation showed similarity between *C. intybus* var. *foliosum* and *C. endivia* var. *endivia*, categorizing them into a single subgroup.

**Figure 10:**
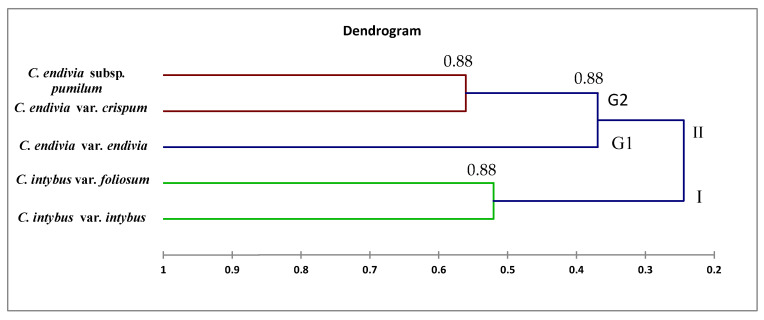
Dendrogram of the relationships between the five taxa of *Cichorium* based on the combined morphological and molecular characters using UPGMA analysis.

The dataset was investigated using clustering analysis to identify and differentiate the taxa. The cluster analysis (Figure 10) showed that species were categorized into two major clusters, and Cluster II was further split into two groups. The first one consisted of two taxa—*C. intybus* var. *intybus* and *C. intybus* var. *foliosum.* Cluster II comprised three taxa and was further divided into two groups: Group 1 contained one taxon *C. endivia* var. *endivia,* while Group 2 had two taxa—*C. endivia* subsp. *pumilum* and *C. endivia* var. *crispum*.

Notably, the derived results are linked to those presented by morphological attributes. It seems likely that there is a significant correlation between *C. endivia* subsp. *pumilum* and *C. endivia* var. *crispum* and between *C. intybus* var. *intybus* and *C. intybus* var. *foliosum*. This may be due to the structure of taxon’s genotype or the effect of evolution naturally.

The correlation matrices possessed a broad sense of similarity (Table 4). The lowest similarity was between the taxon 1 (*C. intybus* var. *intybus*) and 4 (*C. endivia* var. *crispum*) (48% for SCoT, 55% for RAPD, and 57% for all markers, respectively) and between taxon 1 (*C. intybus* var. *intybus*) and 5 (67%) for the ISSR. The highest similarity was between the Object 1 (*C. intybus* var. *intybus*) and 2 (*C. intybus* var. *foliosum*) (88%) for RAPD, the taxon 2 (*C. intybus* var. *foliosum*) and 3 (*C. endivia* var. *endivia*) (86%) for ISSR, the objects 1 (*C. intybus* var. *intybus*) and 2 (*C. intybus* var. *foliosum*) (77%) for the combination, and the Object 4 (*C. endivia* var. *crispum*) and 5 (*C. endivia* subsp. *pumilum*) (73%) for SCoT.

### 2.4. Molecular Distance and Variance Decomposition

The distance to centroids (Table 5) and the variance decomposition (Table 6) of taxa’s classes accounted more in the classification. All taxa were grouped into three classes, as in Figure 8. All markers revealed that *C*. *intybus* var. *intybus* and *C*. *intybus* var. *foliosum* dropped into class (I), *C. endivia* var. *endivia* categorized into class (II) only, and the *C. endivia* var *crispum* and *C. endivia* subsp. *pumilum* formed in class (III); except that the ISSR results showed a different pattern. The ISSR showed that the first class (class I) has *C. intybus* var. *intybus* only, the second (class II) has *C. intybus* var. *foliosum* and *C. endivia* var. *endivia*, and the third class (class II) includes the rest. The variance within the first class was 17.5, and the distance to centroids was 2.96 between *C*. *intybus* var. *intybus* and *C*. *intybus* var. *foliosum*, while the third class recorded a variance of 21.00, and the distance to the centroids was 3.24 between *C. endivia* var *crispum* and *C. endivia* subsp. *pumilum*. The second class, between *C. intybus* var. *foliosum* and *C. endivia* var. *endivia,* coming from ISSR showed an average variance of 3.50 and a distance to the centroids of 1.32, whereas the third class recorded an average variance of 5.50 and a distance of 1.66.

The variance decomposition within and between classes for the optimal classification through three markers, is given in Table 5, the variance averaged 69% within the class, and 31% between classes for the combined markers. Markers showed that the variance within class recorded 60%, 63%, and 66% for RAPD, ISSR, and SCoT, respectively; whereas the variance between classes was 34.43%, 36.62%, and 40.34% for SCoT, ISSR, and RAPD, respectively.

### 2.5. Detrended Canonical Correspondence Analysis (DCCA)

**Figure 11:**
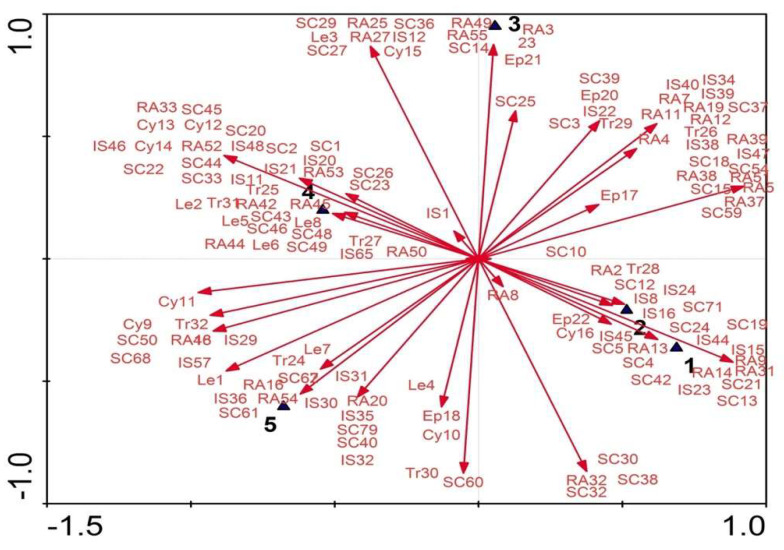
DCCA Triplot of the distribution of the studied taxa and their relationships to morphological and molecular characters. Taxa: 1− *C. intybus* var. *intybus*, 2− *C. intybus* var. *foliosum*, 3− *C. endivia* var. *endivia*, 4− *C. endivia* var. *crispum*, 5− *C. endivia* subsp. *pumilum*. Characters: Le− leaf, Cy− Cypsela, Ep− epidermal, Tr−trichome, SC− SCoT, RA− RAPD, and IS− ISSR.

The DCCA triplot presented in Figure 11 shows the distribution of the studied taxa and their relationships to morphological and molecular characters. *Cichorium intybus* var. *intybus* and *Cichorium intybus* var. *foliosum* were characterized by a short pappus length and stomata associated with an absence of middle lamella. These taxa were also differentiated with SCoT bands (4, 5, 13, 19, 21, 24, 42, and 71), ISSR bands (15, 16, 23, 24, 44, and 45), and RAPD bands (9, 13, 14, and 31) (Figure 11). *Cichorium endivia* var. *endivia* is distinguished by the absence of stephanocytic stomata and reticulate stomatal sculpture. This was also separated by SCoT (14) and RAPD (3, 49, and 55) bands. *Cichorium endivia* var. *crispum* is characterized by a pinnately lobed leaf type, an obtuse leaf apex, a green-colored midrib, absence of radical leaves, a ruminate sulcate surface pattern of cypsela, slightly raised anticlinal walls, and slightly concave periclinal walls. This was also differentiated by SCoT (20, 22, 26, 33, 44, 45, 46, 48, 49, 52, and 53), and RAPD (33, 42, 44, 45, 52, and 53) bands. *Cichorium endivia* subsp. *pumilum* was distinguished by SCoT (61 and 67), ISSR (30 and 36), and RAPD (16 and 54) bands.

## 3. Discussion

The morphological characteristics studied in this research, such as cypsela and pappus, using scanning electron micrographs, have already provided valuable information for specific genera [41,42]. The five taxa of *Cichorium* showed morphological differences. Identification and classification between taxa depend mostly on morphological characteristics. The studied taxa have a wide range of variations in characteristics associated with leaves (shape, type, texture, margin, and apex), cypselae (shape, color, and surface pattern), and trichomes (glands). *Cichorium intybus* is characterized by a spathulate leaf shape, a cypsela sulcate papillate surface pattern, a raised anticlinal wall, a concave periclinal wall, and a pappus type with scabrous scales. *Cichorium endivia* is characterized by having a leaf shape, simple or pinnately lobed, a slightly raised anticlinal wall, a slightly concave periclinal wall, and a pappus type with paleaceous scales to form a crown. The examined taxa demonstrated variations in their morphological features, such as leaf morphology, cypsela, and trichome characteristics. The five taxa had stomata on both surfaces, particularly the abaxial epidermis. Although they are very similar due to their morphological characteristics, *C. intybus* and *C. endivia* have always been categorized into two different species, morphologically described by Kiers et al. [43]. Kiers et al. [7] described *C. intybus* and *C. endivia*, the two cultivated and most known species, using an integration of morphological features with molecular approaches. Our results are in agreement with the view of Kiers et al. [7,43] and Raulier et al. [12].

Genetic identification of *Cichorium* using PCR-based molecular markers (RAPD, ISSR, and SCoT) enabled the detection of polymorphism to reveal the relationships between the studied taxa. Genetic analysis is crucial for managing the entire classification and identification, as well as genetic improvement [44]. The efficiency of a marker for discriminating species depends mainly upon the resultant polymorphism [22,24,45]. Markers of ISSR and SCoT have the potential to reveal polymorphism and offer a higher capacity for the determination of intra- and inter-genomic variations than other arbitrary primers such as RAPDs [21,26,46]. The difference in resolution resulting from RAPDs and ISSRs can be explained by the fact that different positions of the genome are targeted by the two-marker techniques [21]. Therefore, the ability to reveal genetic variability among and within species is directly related to the detected percentage of polymorphism using each marker than the technique employed. Similar conclusions were obtained by Mahdy [26] on lettuce and Jew’s mallow and Gupta et al. [46] on Jatropha. Kiers et al. [7] studied endive and chicory cultivars using diagnostic AFLP markers in *Cichorium* species. Our results are in general agreement with several previous studies [28,29,30,31].

Dendrograms, as illustrated in Figure 8, Figure 9 and Figure 10, have shown a correspondence between molecular markers and morphological attributes. The differences among the dendrograms generated by markers could be partially explained by the different number of PCR products analyzed, as presented in Table 6. This highlights the importance of higher genome coverage and loci number in estimating the genetic relationships among the tested taxa. Loarce et al. [47] obtained similar findings in barley. This could also be explained by the low reproducibility of RAPD markers [48]. The differences in the clustering pattern of genotypes using RAPD and ISSR markers may be attributed to the differences in marker reproducibility and genome coverage among the two tested markers; this highlights the importance of loci number and their genome coverage in obtaining reliable assessments of genetic correlations among taxa [21,22,47]; this may be helpful in genetic diversity, classification, taxonomy, systematics, and evolutionary biology [1,49,50]. Our results showed that the genetic identification and classification depend mainly on the efficiency of a marker [26,51].

Markers had categorized by variance decomposition *C. intybus* var. intybus and *C. intybus* var. *foliosum* into a group and *C. endivia* var. *crispum* and *C. endivia* subsp. *pumilum* into a group except those revealed by ISSR showing *C. intybus* var. foliosum and *C. endivia* var. *endivia* into a group and *C. endivia* subsp. *pumilum* and *C. endivia* var. crispum into a group, as shown in Table 4. That may be due to the variance in the resolution of ISSRs and targeting different loci of the genome by these techniques [21,26,51]. Earlier microsatellite markers could not differentiate between the species [52]. Additionally, it may be due to the makeup of taxon’s genotype or the effect of evolution naturally.

The DCCA analysis of morphological and molecular characteristics revealed that *Cichorium intybus* var. *intybus* is related to *C. intybus* var. *foliosum*, while *C. endivia* var. *endivia* is related to *C. endivia* var. *crispum* and *C. endivia* subsp. *pumilum.* These results are consistent with those of Kiers [11] and Kiers et al. [43]. In earlier publications, it has been argued that the Egyptian chicory was classified into *C. intybus* [38,39,40]. The integrated approaches supports a better authentication and verification of the species and taxa. According to the results of this research we argue that the Egyptian chicory is classified into *C. endivia* subsp. *pumilum.*

## 4. Materials and Methods

### 4.1. Plant Materials

Cypselae of *Cichorium* species were obtained from the collection maintained by Vegetable Production Research Department, Agricultural Research Center (ARC), Giza, Egypt. Five taxa of the genus *Cichorium* were used in this study. Seeds and herbarium specimens of *Cichorium* taxa, *Cichorium intybus* var. *intybus*, *C. intybus* var. *foliosum*, *C. endivia* var. endivia, *C. endivia* var. *crispum*, and *C. endivia* subsp. *pumilum* were identified under the authority of the Herbarium, Botany Dept., National Gene Bank (NGB), Agricultural Research Center (ARC), Giza, Egypt.

### 4.2. Germination

Light intensities at mid-canopy were kept at approximately 400 µmols m^2^s^−1^ in the growth chambers for germination. A photoperiod was adjusted at 16 h of light and 8 h of darkness using fluorescent and incandescent lights. A daytime temperature of 23 °C and a nighttime temperature of 18 °C were maintained using chart recorders. The relative humidity was maintained at approximately 50%. First, the seed surface was sterilized using a solution of 5% sodium hypochlorite for 10 min and rinsed several times with sterile distilled water. Next, 10 seeds of each taxon were replicated in fours on a blotter, to which 10 mL of the test solution was added. Seeds germinated in the growing media containing peat moss, sandy soil, and perlite (1:1:1) under growth chamber conditions. Outer leaves at the basal nodes were taken for macromorphological and micromorphological analysis.

### 4.3. Microscopy

#### 4.3.1. Scanning Electron Microscopy (SEM)

Cypselae and leaves of each *Cichorium* taxon were separately cut into small sections of 4–8 mm long. Glutaraldehyde solution (6%, pH 7.3) was used to fix the sections for 12 h [53]. These were then rinsed with a 0.05 M C_2_H_6_AsNaO_2_ (sodium cacodylate) buffer (pH 7.5) and rinsed with distilled water. This was then dehydrated with gradual ethanol concentrations (10–100%) for 20 min for each concentration. A Hitachi HCP-2 critical point dryer was used to dry the samples, which were then mounted onto aluminum stubs with carbon-coated sputtering (Elko IB-3 Ion Coater). JEOL (JSM-6390 LV) SEM, Model JEOLJSM- 5500 LV was used to examine the samples. As in SEM, both fixation and dehydration procedures were conducted using energy-dispersive X-ray spectroscopy (EDX). NORAN System SIX software (V.1.8) was used for photomicrographs digitally taken at the Electron Microscopy Unit at the National Research Centre, Dokki, Egypt. The terminology for the cypsela, abaxial epidermis, and stomata used in this study follows Barthlott [54], Garg and Sharma [55], Mukherjee and Nordenstam [56], and Dilcher [57]. In addition, all morphological characters regarding the leaf, cypsela, and trichomes were documented to create classical keys.

#### 4.3.2. Light Microscopy

A piece of the middle part of the leaf (1 cm^2^) from each plant was taken and dehydrated in a series of ethanol concentrations (50–100%). The specimens were then embedded in paraffin wax (m.p. 58–61 °C) by xylol (solvent), sectioned at 15 μm on a Jung PM 2045 rotary microtome, and mounted on glass slides with egg albumen (adhesive agent). The wax was dissolved in xylol, and the slides were stained using light green and safranin. Using Canada balsam (mounting agent), permanent slides were prepared [58,59]. A digital camera was used to capture the photomicrographs.

### 4.4. Morphology

Twenty eight traits are presented including thirteen attributes of leaves and fifteen attributes of cypsela; the traits were estimated from approximately ten healthy plants from each taxon and are presented according to the relevant terminology [60].

### 4.5. DNA Extraction

The gDNA extraction was performed by the manufacturer’s instructions of Zymo extraction kit (Zymo Research, Inc., Irvine, CA, USA). The DNA purity and quantity were checked via nanodrop and then stored for PCR analysis. The integrity of gDNA was verified by agarose gel electrophoresis in a 1% agarose/1 × TAE gel containing 1 × Sybr^®^ Safe DNA gel stain (Life Technology, Carlsbad, CA, USA). The isolated genomic DNA samples were diluted to 10 ng/μL. Then, good-quality gDNA samples were used for PCR amplification.

### 4.6. PCR-Based Markers

Three PCR-based markers were used for providing more information on *Cichorium*’s genetic relatedness which are random amplified polymorphic DNA (RAPD), Inter Simple Sequence Repeats (ISSR), and start codon target (SCoT), as given in Table 3. RAPD-PCR amplification was carried out with ten synthesized random 10-mer arbitrary primers (Operon Biotechnologies, Inc., Ebersberg, Germany). The procedure was done as detailed in Williams et al. [20] and Williams and St Clair [30] with minor modifications. The amplification by ISSR to detect polymorphisms among accession for both species was carried out as described by Yang and Park [61] with slight modifications. The procedure of SCoT was designed by describing of Collard and Mackill [62], which synthesized by Operon Biotechnologies, Inc., GmbH, (Cologne, Germany).

The PCR reactions were performed in a 25 μL reaction mixture that contained 25 ng of template DNA, 0.2 μM dNTPs, 1 µM of each tested primer, 1.5 mM MgCl_2_, 1× PCR buffer, and 1 U of Go-Taq Flexi polymerase. In the initial denaturation cycle, the PCR program was adjusted at 94 °C for 5 min, then followed by 35 cycles. Each cycle involved 94 °C for 1 min, varying annealing temperature for each primer (Table 3) for 1 min, then 72 °C for 90 s, and 72 °C for 7 min in the final step for extension. The PCR amplification reaction products (Amplicons) were resolved in 1.0–1.5% agarose gel that contained ethidium bromide (0.5 µg/mL) in 1× TBE as a running buffer. A 100 bp plus DNA Ladder was used as molecular size standards.

PCR products were run on agarose gel in 1% agarose × TAE gel containing 1 × Sybr^®^ Safe DNA gel stain (Life Technology, Carlsbad, CA, USA) at 100 V for 30 min. Gels were visualized and photographed with a Gel Doc^TM^ XR+ System with Image Lab^TM^ Software (Bio-Rad^®^). Amplicon banding profiles were scored as present (1) or absent (0) in a binary matrix based on standard markers using Alpha Ease FCTM (version 4.0.1) software.

### 4.7. Statistical Analysis

Similarity for a binary matrix was estimated according to the Jaccard coefficient [63]. The dendrogram generated the Un-weighted Pair Group Method algorithms with Arithmetic (UPGMA) averages according to Nei and Li [64] to determine the genetic relationship among taxa. In addition, a Detrended Canonical Correspondence Analysis (DCCA) was performed using CANOCO V. 4.5 and CanoDraw V. 4.1. The dataset was entered into SPSS (version 14.0), as well as the add-in packages of StatistiXL (Kovach Computing Service 2013, version 1.8: http://www.xlstat.com (last accessed on 7 February 2022) and GenAlEx Genetic Analysis (version 6.5) in Microsoft Excel [65,66].

## 5. Conclusions

The integration of various identification methods, including macro- and micro-morphological and molecular characterization, plays a chief role in the identification and authentication of plant genera, species, and taxa. Although measuring the variability of morphological attributes does not provide extensive information, it can still be helpful for breeding and improvement programs. This is especially important in changing climates, including the Mediterranean region. The existence of these distinct taxa and their phylogenetic relationships were assessed and confirmed in this study using the phenological, and molecular markers, and phylogenetic methodologies. The approaches used in this study proved that these tools are helpful for phylogenetic studies at the species level or higher taxonomic ranks in the genus *Cichorium*.

We report that the leaf shape and type, the cypsela anticlinal and periclinal walls, pappus bristles, the epidermal cell wall, and the stomatal type were the most critical characteristics in the construction of the dichotomous indented key for *Cichorium* taxa. PCR-based identification confirmed these results revealed through the phylogenic tree. We observe that the integration of phenological and genetic characterization is helpful in the authentication of *Cichorium* taxa. Further research could focus on intra-species variability, diversity, and genetic differences among individuals of the *Cichorium* taxa. Despite the success of the molecular marker approach, we recommend the use of supplemental techniques such as DNA barcoding for improved classification and identification, along with more core and base collections of the studied plant materials. Our observations suggest that these taxa have the potential to provide rich genetic resources for further research in genetic biodiversity, conservation, and plant breeding programs. The chicory genetic assembly among different countries in the Mediterranean region visibly diverges with a firm amount of gene flow. The PCR-based markers could be utilized for analyzing genetic relationships and taxonomies of *Cichorium* taxa. Finally, it could be concluded that the Egyptian chicory belongs to the *C. endivia* subsp. *pumilum*.

## Figures and Tables

**Table 1:** Morphological characters of leaf and cypsela of *Cichorium*.

Attributes		1	2	3	4	5
Leaf	Shape	Spathulate	Spathulate	spathulate	obovate	oblancelate
	Type	Simple	Simple	simple	pinnately lobed	simple
	Petiole	Present	Present	absent	absent	present
	Margin	Dentate	fine dentate	dentate	dentate	fine dentate
	Apex	Obtuse	Obtuse	obtuse	obtuse	obtuse
	Midrib	Distinct	Distinct	distinct	indistinct	distinct
	Midrib color	White	White	white	green	white
	Lateral venation	Distinct	not distinct	distinct	indistinct	indistinct
	Base	Symmetrical	Symmetrical	symmetrical	symmetrical	symmetrical
	Venation	Reticulate	Reticulate	reticulate	reticulate	reticulate
	Surface	Glabrous	Glabrous	glabrous	glabrous	glabrous
	Stipule	Stipulate	Stipulate	stipulate	stipulate	stipulate
	Radical leaf	Present	Present	present	absent	present
Cypsela	Color	creamy shiny to black with creamy	creamy shiny to dark brown with creamy	creamy shiny to dark brown with creamy	pale creamy shiny to creamy with black point	creamy shiny with few dark brown point
	Shape	Oblong	oblong obovate	oblong	oblong	oblong obovate
	Length (mm)	2.6–3	2.6–3	2.3–3	2.4–3	1.7–2.25
	Width (mm)	0.96–1.2	1.15–1.3	1.2–1.3	1.1–1.5	1–1.3
	Size (L × W) (mm^2^)	2.8 × 1.1	2.8 × 1.2	2.7 × 1.25	2.7 × 1.3	2.4 × 1.1
	Texture	smooth with grooves	smooth with grooves	smooth with grooves	smooth with grooves	smooth with grooves
	Hilum shape	circular	circular	circular	circular	circular
	Surface pattern	sulcate papillate	sulcate papillate	weak sulcate papillate	ruminate- sulcate	weak ruminate sulcate papillate
	Anticlinal wall shape	Straight	Straight	straight	straight	straight
	Anticlinal walls	Raised	Raised	slightly raised	slightly raised	slightly raised
	Periclinal walls	Concave	Concave	slightly concave	slightly concave	slightly concave
	Gonal (number)	3–4	4	4–5	4–5	4–5
	Pappus bristles (type)	scabrous scales	scabrous scales	paleaceous scales to form crown	paleaceous scales to form crown	paleaceous scales to form crown
	Pappus persistence	Persistence	Persistence	persistence	persistence	persistence
	Pappus length	Short	Short	Long	Long	short

1- *Cichorium intybus* var. *intybus*, 2- *Cichorium intybus* var. *foliosum*, 3- *Cichorium endivia* var. *endivia*, 4- *Cichorium endivia* var. *crispum*, and 5- *Cichorium endivia* subsp. *pumilum*.

**Table 2:** Micro-morphological characters of epidermis and trichome in the studied taxa.

Attribute				1	2	3	4	5
Epidermal	Epidermal cell wall: (1) Straight. (2) Sinuous.			2	2	1	2	1
	Stomata leveling: (1) Superficial. (2) Semi-depressed. (3) Depressed.			3	1	2	2	1
	Stomata types		Anisocytic stomata: (1) Present. (2) Absent.	1	2	1	1	2
			Anomocytic stomata: (1) Present. (2) Absent.	1	1	1	1	1
			Tetracytic stomata: (1) Present. (2) Absent.	1	2	2	1	1
			Stephanocytic stomata: (1) Present. (2) Absent.	1	1	2	1	1
			Associated stomata with middle lamella: (1) Present. (2) Absent.	2	2	1	2	1
	Sculpture: (1) Striate. (2) Reticulate.			1	1	2	1	1
Trichome	Non-glandular	Papillae: (1) Present. (2) Absent.		1	2	1	2	2
		Multicellular hairs with heterogeneous cells: (1) Present. (2) Absent.		2	1	2	2	2
		Multicellular hairs with homogeneous cells: (1) Present (2) Absent		2	2	2	2	1
		Shaggy hair: (1) Present (2) Absent		1	2	1	2	1
	Glandular	Multicellular uniseriate stalk and unicellular head with homogeneous cells: (1) Present (2) Absent		2	2	2	1	2
		Multicellular uniseriate stalk and unicellular head with heterogeneous cells: (1) Present (2) Absent		1	2	2	1	1
		Multicellular uniseriate stalk and bicellular head with homogeneous cells: (1) Present (2) Absent		2	2	1	2	2
		Multicellular uniseriate stalk and bicellular head with homogeneous cells: (1) Present (2) Absent		2	1	2	2	2
	Ornamentation: (1) Verrucose. (2) Smooth. (3) Ttuberculate & warty. (4) Smooth & warty			1	2	2	3	4

1- *C. intybus* var. *intybus*, 2- *C. intybus* var. *foliosum*, 3- *C. endivia* var. *endivia*, 4- *C. endivia* var. *crispum*, and 5- *C. endivia* subsp. *pumilum*.

**Table 3:** Primer names, sequences (5’-3’), and their annealing temperatures (T_a_), total, monomorphic, polymorphic, and unique bands, and polymorphism (%) for the three markers used in the study.

Marker	Primer	Sequence	Tm (℃)	TB	MB	PB	UB	%P
RAPD	OPA7	GAAACGGGTG	32	6	2	3	1	66.67
	OPA9	GGGTAACGCC	34	4	1	2	1	75.00
	OPB5	TGCGCCCTTC	37	5	1	2	2	80.00
	OPC4	CCGCATCTAC	32	2	1	1	0	50.00
	OPD5	TGAGCGGACA	38	7	4	2	1	42.86
	OPA2	TGCCGAGCTG	43	7	4	3	0	42.86
	OPC5	GATGACCGCC	40	4	2	2	0	50.00
	OPC8	TGGACCGGTG	42	6	3	3	0	50.00
	OPA10	CTGCTGGGAC	32	7	3	4	0	57.14
	OPA18	AGGTGACCGT	32	7	0	7	0	100.0
Total				55	21	29	5	61.82
ISSR	HB 8	(ga)6 gg	44	4	3	1	0	25.00
	HB 12	(cac)3 gc	44	3	3	0	0	0.00
	HB 13	(gag)3 gc	44	7	4	2	1	42.86
	ISSR 1	cac (tcc)5	53	5	3	2	0	40.00
	ISSR 3	tgta (ca)7	53	7	2	3	2	71.43
	UBC888	tac (ca)7	47	8	1	6	1	87.50
	UBC807	(ag)8 t	38	9	4	2	3	55.56
	ISSR16	cgtc (ac)7	44	7	4	3	0	42.86
	ISSR 35	tcga (ca)7	53	6	5	1	0	16.67
	UBC834	(ag)8 ct	44	5	4	1	0	20.00
Total				61	33	21	7	45.90
SCoT	SCoT-02	ACCATGGCTACCACCGGC	50	8	2	4	2	75.00
	SCoT-03	ACGACATGGCGACCCACA	57	10	1	9	0	90.00
	SCoT-04	ACCATGGCTACCACCGCA	56	4	0	4	0	100.0
	SCoT-05	CAATGGCTACCACTAGCG	55	10	2	7	1	80.00
	SCoT-06	CAATGGCTACCACTACAG	55	7	1	5	1	85.71
	SCoT-09	ACAATGGCTACCACTGCC	55	7	3	4	0	57.14
	SCoT-11	ACAATGGCTACCACTACC	50	8	4	3	1	50.00
	SCoT-12	CAACAATGGCTACCACCG	61	6	3	3	0	50.00
	SCoT-13	ACCATGGCTACCACGGCA	61	6	5	1	0	16.67
	SCoT-14	ACCATGGCTACCAGCGCG	55	4	3	1	0	25.00
Total				70	24	41	5	65.71
Overall Total				186	78	91	17	58.06

Total bands (TB), monomorphic bands (MB), polymorphic bands (PB), unique bands (UB), percentage of polymorphism (%P).

**Table 4:** Proximity matrix using Jaccard coefficient among the taxa of *Cichorium*.

Taxa			1	2	3	4	5
ISSR	1	*C. intybus* var. *intybus*	1.00				
	2	*C. intybus* var. *foliosum*	0.76	1.00			
	3	*C. endivia* var. *endivia*	0.71	0.86	1.00		
	4	*C. endivia* var. *crispum*	0.70	0.72	0.77	1.00	
	5	*C. endivia* subsp. *Pumilum*	0.67	0.68	0.73	0.79	1.00
SCoT	1	*C. intybus* var. *intybus*	1.00				
	2	*C. intybus* var. *foliosum*	0.71	1.00			
	3	*C. endivia* var. *endivia*	0.53	0.61	1.00		
	4	*C. endivia* var. *crispum*	0.48	0.53	0.65	1.00	
	5	*C. endivia* subsp. *Pumilum*	0.55	0.60	0.61	0.73	1.00
RAPD	1	*C. intybus* var. *intybus*	1.00				
	2	*C. intybus* var. *foliosum*	0.88	1.00			
	3	*C. endivia* var. *endivia*	0.66	0.69	1.00		
	4	*C. endivia* var. *crispum*	0.55	0.58	0.60	1.00	
	5	*C. endivia* subsp. *Pumilum*	0.58	0.60	0.59	0.68	1.00
Combined	1	*C. intybus* var. *intybus*	1.00				
	2	*C. intybus* var. *foliosum*	0.77	1.00			
	3	*C. endivia* var. *endivia*	0.63	0.72	1.00		
	4	*C. endivia* var. *crispum*	0.57	0.61	0.68	1.00	
	5	*C. endivia* subsp. *Pumilum*	0.60	0.63	0.65	0.73	1.00

Colors range from lowest value (red) through medium value (yellow) to highest value (green).

**Table 5:** Distances to centroids between the classes of taxa.

Class		I	II	III
RAPD	No. of taxa	2	1	2
	Within-class variance	2.5000	0.0000	8.0000
	Minimum distance to centroid	1.1180	0.0000	2.0000
	Average distance to centroid	1.1180	0.0000	2.0000
	Maximum distance to centroid	1.1180	0.0000	2.0000
ISSR	No. of taxa	1	2	2
	Within-class variance	0.0000	3.5000	5.5000
	Minimum distance to centroid	0.0000	1.3229	1.6583
	Average distance to centroid	0.0000	1.3229	1.6583
	Maximum distance to centroid	0.0000	1.3229	1.6583
SCoT	No. of taxa	2	1	2
	Within-class variance	8.5000	0.0000	7.5000
	Minimum distance to centroid	2.0616	0.0000	1.9365
	Average distance to centroid	2.0616	0.0000	1.9365
	Maximum distance to centroid	2.0616	0.0000	1.9365
Combined	No. of taxa	2	1	2
	Within-class variance	17.5000	0.0000	21.0000
	Minimum distance to centroid	2.9580	0.0000	3.2404
	Average distance to centroid	2.9580	0.0000	3.2404
	Maximum distance to centroid	2.9580	0.0000	3.2404

**Table 6:** Variance decomposition for the optimal classification.

Class		Absolute	Percent
RAPD	Within class	5.25	59.66%
	Between classes	3.55	40.34%
ISSR	Within class	4.5	63.38%
	Between classes	2.6	36.62%
SCoT	Within class	8	65.57%
	Between classes	4.2	34.43%
Combined	Within class	19.25	69.00%
	Between classes	8.65	31.00%

## Data Availability

Not applicable.
